# Design, Build, and Initial Testing of a Portable Methane Measurement Platform

**DOI:** 10.3390/s25071954

**Published:** 2025-03-21

**Authors:** Stuart N. Riddick, John C. Riddick, Elijah Kiplimo, Bryan Rainwater, Mercy Mbua, Fancy Cheptonui, Kate Laughery, Ezra Levin, Daniel J. Zimmerle

**Affiliations:** 1 Department of Science, Engineering and Aviation, University of the Highlands and Islands Perth, Crieff Road, Perth PH1 2NX, UK; 2 Methane Emission Technology Evaluation Center (METEC), Energy Institute, Colorado State University, Fort Collins, CO 80524, USA; elijah.kiplimo@colostate.edu (E.K.); bryan.rainwater@colostate.edu (B.R.); mercy.mbua@colostate.edu (M.M.); fancy.cheptonui@colostate.edu (F.C.); kate.laughery@colostate.edu (K.L.); ezra.levin@colostate.edu (E.L.); dan.zimmerle@colostate.edu (D.J.Z.); 3 Independent Researcher, Lockerbie DG11 2BE, UK; john_riddick@hotmail.com

**Keywords:** methane, concentration, quantification

## Abstract

The quantification of methane concentrations in air is essential for the quantification of methane emissions, which in turn is necessary to determine absolute emissions and the efficacy of emission mitigation strategies. These are essential if countries are to meet climate goals. Large-scale deployment of methane analyzers across millions of emission sites is prohibitively expensive, and lower-cost instrumentation has been recently developed as an alternative. Currently, it is unclear how cheaper instrumentation will affect measurement resolution or accuracy. To test this, the Wireless Autonomous Transportable Methane Emission Reporting System (WATCH_4_ERS) has been developed, comprising four commercially available sensing technologies: metal oxide (MOx,), Non-dispersion Infrared (NDIR), integrated infrared (INIR), and tunable diode laser absorption spectrometer (TDLAS). WATCHERS is the accumulated knowledge of several long-term methane measurement projects at Colorado State University’s Methane Emission Technology Evaluation Center (METEC), and this study describes the integration of these sensors into a single unit and reports initial instrument response to calibration procedures and controlled release experiments. Specifically, this paper aims to describe the development of the WATCH_4_ERS unit, report initial sensor responses, and describe future research goals. Meanwhile, future work will use data gathered by multiple WATCH_4_ERS units to 1. better understand the cost–benefit balance of methane sensors, and 2. identify how decreasing instrumentation costs could increase deployment coverage and therefore inform large-scale methane monitoring strategies. Both calibration and response experiments indicate the INIR has little practical use for measuring methane concentrations less than 500 ppm. The MOx sensor is shown to have a logarithmic response to methane concentration change between background and 600 ppm but it is strongly suggested that passively sampling MOx sensors cannot respond fast enough to report concentrations that change in a sub-minute time frame. The NDIR sensor reported a linear change to methane concentration between background and 600 ppm, although there was a noticeable lag in reporting changing concentration, especially at higher values, and individual peaks could be observed throughout the experiment even when the plumes were released 5 s apart. The TDLAS sensor reported all changes in concentration but remains prohibitively expensive. Our findings suggest that each sensor technology could be optimized by either operational design or deployment location to quantify methane emissions. The WATCH_4_ERS units will be deployed in real-world environments to investigate the utility of each in the future.

## 1. Introduction

With deadlines to meet climate pledges imminent, international desire to quantify methane emission reductions has increased over the past five years [1,2]. While top-down quantification methods, such as satellite or tall tower approaches [3,4], can be used to generate relatively large-scale emission estimates (areas of tens of kilometers), near-field methods are necessary to quantify site-scale emissions (areas of tens of meters) [5,6,7]. Near-field methods typically use methane concentrations measured downwind coupled with meteorological observations to infer an emission rate using dispersion approaches that follow Gaussian, Lagrangian, and Navier–Stokes modeling approaches [8,9,10,11,12,13].

The main shortcoming of near-field methane quantification is the cost of instrumentation sensitive enough to measure downwind methane concentrations, typically less than 50 ppm [14,15,16]. Since the late 1980s, cavity-based optical analyzers have been considered essential for measuring methane concentrations in this range [17,18]. However, these instruments are expensive (>USD 30,000), and exclusively using optical cavity analyzers remains prohibitively expensive as there are hundreds of thousands of methane sources that require quantification, e.g., oil and gas production pads, landfills, wastewater treatment sites, concentrated animal feeding operations (CAFOs), and dairy farms.

To address this shortcoming, lower-cost sensors are being repurposed [19,20] or developed [21] but it is currently unclear if these sensors can replace optical cavity analyzers as a compromise between cost, response time, and accuracy. As part of the US Department of Energy’s (DoE) project to decarbonize natural gas resources, multiple low-cost sensing technologies that are currently commercially available are being evaluated as part of the US Department of Energy-funded redevelopment of Colorado State University’s Methane Emission Technology Evaluation Center (METEC). To investigate if lower-cost technologies are accurate/sensitive enough to replace higher-cost analyzers, four methane-sensitive technologies are co-located within an IP-69 rated box, powered using solar with and data transmitted via the mobile network and used to measure downwind methane concentrations. The four technologies are metal oxide (MOx) sensors (USD 15 per sensor), a non-dispersive infrared (NDIR) sensor (USD 500 per sensor), an integrated infrared (INIR) sensor (USD 300 per sensor), and a tunable diode laser absorption spectrometer (TDLAS) (USD 15,000 per sensor). Henceforth, this package will be referred to as the Wireless Autonomous Transportable Methane Emission Reporting System (WATCH_4_ERS).

The WATCH_4_ERS unit was developed over the past five years and has leveraged hitherto unpublished research methods developed by the METEC research group that has advanced methane sensor integration. The data from multiple WATCH_4_ERS units will be used to investigate the long-term utility of the instrumentation, but the aim of this paper is to describe the WATCH_4_ERS units’ design/build and report a sample of the unit’s data output as a preview of what will become available during long-term deployments during the DoE-funded controlled release experiments at aerodynamically complex oil and gas production and transportation facilities. As such, we feel it would not be useful to use the data collected during the very short experiment described in this paper to define each sensor’s characteristics, e.g., response time or signal-to-noise ratios, and these properties will not be calculated as part of the study presented here. Here, we acknowledge that novel methane-sensing devices that could be very applicable to this project are being developed using 2D materials, nanocomposites, or interband-cascade light-emitting technology [22,23,24,25,26]. However, these were not considered for use in this project as they are not commercially available and therefore do not come with manufacture quality or supply guarantees.

MOx sensors typically use a tin dioxide (SnO_2_) sensing layer that reacts with detected gases. The detection mechanism relies on the change in electrical conductivity through the oxide layer in the presence of methane which is a reducing gas. An absorbance of oxygen molecules occurs on the surface of SnO_2_, forming O^2−^ ions and leading to electron capture creating a depletion layer at the surface of the SnO_2,_ increasing its resistance [27,28]. Methane interacts with the adsorbed oxygen ions thus releasing the trapped electrons back into the SnO_2_ conduction band, thereby decreasing its resistance. The reduction in resistance is proportional to the methane concentration. Over the last five years, metal oxide sensors have increasingly been used to detect and quantify emissions from oil and gas production infrastructure [6,27,29]. Low-cost MOx sensors are of great interest as the quantification of emissions from each emitting site (landfill, oil and gas production, wastewater, etc.) would require at least four sensors [30]. Lower-cost sensors would make the financial burden of measurement more manageable; however, it is unclear how responsive these sensors are [31].

NDIR multi-gas sensing platforms have been developed to measure methane concentrations, as well as carbon dioxide, nitrous oxide, and water, to sub-ppm mixing ratios [21]. The NDIR uses a multi-spectral and multi-optical path design coupled with three individual wavelength-selective detectors to observe the absorption of species-specific radiation and infer concentrations within an air sample [21,32]. As of 2025, NDIR sensors are relatively new to the methane emission quantification field and it is currently unclear how useful these will be compared to more established instrumentation (MOx and TDLAS).

Similarly, INIR sensors use the absorption of infrared radiation to infer the abundance of methane in a sample of air between 100 ppm and 100% [33]. Infrared light passes through the gas sample chamber, and methane absorbs specific infrared wavelengths. The reduction in infrared intensity at these wavelengths, measured by the detector, is proportional to the methane concentration. This approach ensures high accuracy and selectivity for methane detection, with minimal interference from other gases. As INIR sensors have no heated elements, they are much safer to use around methane sources than MOx sensors and can therefore be installed much nearer to the source of emission [34]. The INIR was included as part of the WATCH_4_ERS instrumentation as it has been reported that fast-responding sensors are able to observe and quantify high-concentration vortices within the plume [35], but it is currently unclear if sensors with higher detection limits, such as the SGX INIR, are fast enough to detect these.

TDLAS uses a laser tuned to the absorption wavelength of methane [36]. The methane concentration in air is calculated from the change in the laser’s intensity as it passes through a sample of air within a measurement cavity [37]. TDLAS instruments have a lower minimum detection limit than MOX, NDIR, and INIR sensors, a higher signal-to-noise ratio, and can instantaneously report changes in methane concentration [17]. The main shortcoming is that the laser absorption inside the cavity can be affected by dust and the accuracy of measurement is reduced by thermal expansion of the cavity. Even though precision and accuracy are lower than optical cavity analyzers, TDLAS instruments have been used in many studies to report methane emissions [38,39,40,41,42]. However, TDLAS instruments are still relatively expensive and it is currently unclear how much the benefits in precision and accuracy outweigh the cost when compared to less expensive measurement options (MOx, NDIR, and INIR).

While there have been studies that have reported the response time of fugitive methane emission detection systems, the raw measurement data remained proprietary to the solution developers and unavailable for analysis [43]. The aim of the WATCH_4_ERS project is to provide data that can be used to 1. better understand the cost–benefit balance of methane sensors, and 2. identify how decreasing instrumentation costs could increase deployment coverage and therefore inform large-scale methane monitoring strategies. This paper describes the development of the WATCH_4_ERS unit which uses four sensing technologies (MOx, NDIR, INIR, and TDLAS) to measure the methane concentrations in air at different upper/lower thresholds, with different levels of precision, and respond at different rates to changes in methane concentrations. Here, we provide a comprehensive guide on the sensor integration of low-cost commercially available products, including all data acquisition software.

## 2. Materials and Methods

The WATCH_4_ERS comprises five individual methane sensors (two MOx sensors, an NDIR sensor, an INIR sensor, and a TDLAS) that stream data to a single laptop PC via a powered USB hub. Data are read into the PC using Python code (version 3.12.9) and data are stored locally. Hardware, software, materials, and build details are presented for each of the sensors below.

### 2.1. Data Acquisition

The code required to run the WATCH_4_ERS can be found at https://github.com/stuartnriddick/AMMMU.git (accessed on 3 March 2025). Three Arduino UNOs are used to interface the MOx sensors, the ’46 Hawk (TDLAS sensor), and the environment monitoring sensor (DHT22) to the data-logging PC through a standard USB port. For the TGS2600 and TGS2611 metal oxide sensors, an Arduino UNO along with an Adafruit ADS1115 ADC interfaces the sensors, and the code “Riddick MOX Arduino Code.ino” controls the sampling of these sensors and the transfer of the data to the PC. The ’46 Hawk sensor does not have any digital output and the data from this instrument is extracted by a technique known as “data sniffing” where code run by the monitoring Arduino interrogates the ’46 Hawk LCD and extracts the values displayed on the LCD. Sniffing the data transmitted to the LCD screen of the ’46 Hawk requires “Hawk Arduino Code.ino” downloaded to the Arduino UNO connected to it. The other two sensors, the INIR and the NDIR, both have serial RS232 interfaces, and these outputs are converted using RS232-to-USB converter modules plugged directly into the USB Hub. The Python code that collects data from all the sensors and writes them to a file is “Ammu Python Code.py”.

### 2.2. Sensors

#### 2.2.1. Metal Oxide (MOx)

Typical metal oxide (MOx) sensor models used in these studies are the Taguchi Gas Sensors (TGS) 2600 (power consumption 210 mW) and 2611 (power consumption 280 mW) models produced by Figaro Engineering Inc. (Osaka, Japan). The TGS2600 uses a heated tin dioxide (SnO_2_) sensing layer that reacts with the detected gases. The detection mechanism relies on the change in electrical conductivity through the SnO_2_ in the presence of methane which is a reducing gas [19,20,27]. The TGS2611 has the same operation basis as the TGS2600 but has been optimized for methane detection by the integration of a heating element to maintain optimal temperature for methane detection [27,28]. Additionally, the TGS2611 features a filter material that selectively permits methane to reach the sensing element while blocking other gases [27,28]. As well as methane, both the TGS2600 and TGS2611 are sensitive to carbon monoxide, iso-butane, ethanol, and hydrogen [44,45].

The metal oxide sensor has a resistance (*R*_0_) in clean air, i.e., air with ambient methane, which becomes lower in the presence of methane (*R_s_*). The ratio of these resistances (*R*_0_/*R_s_*) gives a measure of the methane mixing ratio in air. However, the resistance of the metal oxide sensor is also affected by the air temperature and relative humidity; therefore, the ratio of resistance must be corrected for these factors [19]. A DHT11 sensor was used to measure temperature and relative humidity (as described in Section 2.2.5). Both Figaro TGS sensors, 2600 and 2611, were powered by 5 V LM7805 voltage regulators (Supplementary Materials Figure S1). The voltage output from the TGS sensors was converted using an ADS1115, 16-bit analog-to-digital converter controlled by an Arduino UNO. The output was communicated to the PC laptop via the USB C port integrated into the Arduino UNO. An external 12 Volt DC source powered the data collection package. Pseudo-code that describes the Arduino-based software (Riddick MOX Arduino Code.ino) is presented in Supplementary Material Section S3.1.1 and the actual code is in Supplementary Material Section S3.1.2.

#### 2.2.2. Non-Dispersive Infrared (NDIR)

The K96 sensor (power consumption 500 mW) from Sensair (Delsbo, Sweden) is a compact non-dispersive infrared multi-gas sensing platform that measures methane, carbon dioxide, nitrous oxide, and water, to sub-ppm levels [21]. The K96 uses a multi-spectral and multi-optical path design coupled with three individual wavelength-selective detectors to observe the absorption of species-specific radiation and infer concentrations within an air sample [21,32]. The sensor outputs data on three channels: the LPL channel measuring CO_2_, N_2_O, and CH_4_ (all in ppm); the SPL channel measuring CO_2_ in ppm; and the MPL channel measuring H_2_O in % volume. The K96 and RS232 formatted data were sent to a laptop via Rx/TX pins on an FTDI FT232RL IC using a Python script. Data are updated every second and stored on the laptop’s hard drive. The K96 sensors were co-located to within 5 cm of the TGS2600 and TGS2611 sensors and passively exposed to the air (Supplementary Materials Figure S4).

#### 2.2.3. Integrated Infrared (INIR)

The SGX INIR-ME100 (power consumption 100 mW) sensor (SGX, Katowice, Poland) uses the absorption of infrared radiation to infer the abundance of methane in a sample of air between 100 ppm and 100% [33]. Infrared light passes through the gas sample chamber and methane absorbs specific IR wavelengths. The reduction in infrared intensity at these wavelengths, measured by the detector, is proportional to the methane concentration. The SGX INIR-ME100 directly outputs methane mixing ratio data and data are sent/read to a laptop via Rx/TX pins on an FTDI FT232RL IC using a Python script. Data are updated every second and stored on the laptop’s hard drive. The INIR sensor was co-located to within 5 cm of the other sensors and passively exposed to the air. The SGX INIR-ME100 is sold as a methane-specific sensor.

#### 2.2.4. Tunable Diode Laser Absorption Spectrometer (TDLAS)

Methane mixing ratios were also measured by a Southern Cross Inc. (Norcross, GA, USA) ’46 Hawk tunable diode laser absorption spectrometer (power consumption 1.5 W). The ’46 Hawk is a methane-specific instrument that draws air into the cavity of the handheld unit at a rate of 1.2 lpm and methane mixing ratios are reported every second [46]. The response time of the ’46 Hawk is 2 s and can measure methane concentrations between 1 ppm and 100% gas with a sensitivity of 1 ppm. Gas measurements made by the ’46 Hawk are only displayed on an LCD screen and an Arduino-based interface was designed to capture and decode the displayed measurements. This information was then transferred through an RS232 interface to the data-logging PC. The sampling inlet of the ’46 Hawk was collocated (within 5 cm) with the TGS, INIR, and K96 sensors.

The interface between the ’46 Hawk LCD and the Arduino was accomplished by decoding the digits displayed on a liquid crystal display (LCD) allowing these values to be logged to a PC (Supplementary Material Section S1 Figures S2 and S3). This process does not interrupt measurement and simply monitors the data being transferred between the Hawk and LCD screen in real-time, it is assumed that this is an instantaneous process with no delay between measurement and recording. An Arduino UNO was used to monitor the three ‘46 Hawk LCD control lines (Enable, RS, R/W) and read data transmitted to the four LCD data lines. Data lines were read sequentially until the 16 data segments that characterized the LCD output had been read. These data were then converted to ASCII format and output by the Arduino as a serial string. These string data were then transferred to the PC via RS232 and a USB converter. The connections between the Hawk LCD and the Arduino port were made using an 8-way ribbon cable, the implementation of which required delicate soldering as the separation between the ribbon cable wires was 1.27 mm with each conductor 0.3 mm in diameter. Pseudo-code that describes the Arudino-based software (Hawk Arduino Code.ino) is presented in Supplementary Material Section S3.2.1 and the actual code is in Supplementary Material Section S3.2.2.

#### 2.2.5. Temperature and Relative Humidity

Temperature and relative humidity used to apply correction to the TGS data were measured using a DHT11 sensor and communicated to the laptop via an Arduino UNO.

### 2.3. Operating Procedures

To start data collection, all USB connectors from the individual sensors should be attached to the USB hub and the hub attached to the laptop PC (Supplementary Material Section S1 Figures S1–S3), and sensors installed in a sensor box (Supplementary Material Section S1 Figure S4; Supplementary Material Section S2 Table S1). The Python code “AMMMU_Python_Code.py” should be installed into a separate folder on the C Drive. Before the Python code is run, the assignment of each instrument’s com port should be updated in the code on lines 24 (TGS sensors port), line 25 (DHT11 sensor), line 26 (INIR sensor), line 36 (K96 sensor), and line 82 (’46 Hawk). The ’46 Hawk should be powered up first and alarm set to 10,000 ppm and the volume set to “Off”. The other sensors can then be powered up. The Python module can then be set to run. Data are written to a new daily text file without headers in the folder that contains “AMMMU_Python_Code.py” with the naming format “MULTI_Sensor YYMMDD.txt”. Experimental data written on the same day will be concatenated to the same text file.

### 2.4. Data Files

Data are recorded by the Arduino UNO as a text file in a space-delimited format with 17 columns (Supplementary Material Section S2 Table S2). The main columns of interest for calculating methane concentrations are the time (Column 1; HH:MM:SS), TGS2600 output (Column 2; counts), TGS2611 output (Column 3; counts), K96 LPL channel (Column 5; the sum of CO_2_, N_2_O, and CH_4_ concentrations in ppm), K96 SPL channel (Column 6; CO_2_ concentration in ppm), Hawk output (Column 13, CH_4_ concentration in ppm), INIR output (Column 14; CH_4_ concentration in ppm), DHT 11 relative humidity (Column 16, %), and DHT 11 temperature (Column 17, °C).

### 2.5. Sensor Calibration Method

**Figure 1:**
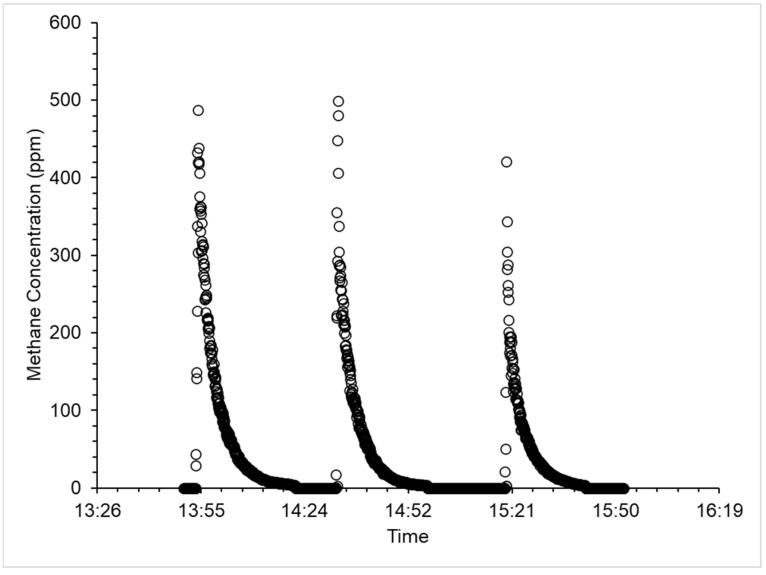
Methane concentrations inside a nonreactive plastic (polyvinyl chloride) cylindrical (diameter 0.35 m, height 0.4 m) chamber at 20 °C and 60% humidity as measured by a ’46 Hawk TDLAS during the calibration experiment. Following a 2 h period of acclimatization, 0.5% methane gas from a calibration gas cylinder (accuracy ± 2%) was injected into the chamber at a rate of 1 g CH_4_ h^−1^ until the TDLAS registered a methane concentration greater than 400 ppm.

The MOx, NDIR, and INIR sensors within the WATCH_4_ERS unit were calibrated by comparing the sensor output to the TDLAS output, following the calibration of the TDLAS using the procedure outlined in the Southern Cross manual [46]. The WATCH_4_ERS unit was placed in a nonreactive plastic (polyvinyl chloride) cylindrical (diameter 0.35 m, height 0.4 m) chamber at 20 °C and 60% humidity. This chamber was not sealed and vented to the atmosphere to reduce any pressure change caused by injecting gas into the chamber. Following a 2 h period of acclimatization, 0.5% methane gas from a calibration gas cylinder (accuracy ±2%) was injected into the chamber at a rate of 1 g CH_4_ h^−1^ until the TDLAS registered a methane concentration greater than 400 ppm. This was repeated three times over a period of 2 h (Figure 1). The MOx, NDIR, and INIR sensors were then calibrated by comparing their output to the TDLAS output and calibration algorithms generated. The MOx voltage output, as measured by the potential divider circuit (Supplementary Material Section S1 Figures S1 and S2, with data capture described in Supplementary Material Section S3.1) was corrected for the effects of temperature and relative humidity [19] and used to calculate the relative resistance of the metal oxide strip (*R_r_*, Supplementary Material Section S4 Equation (S1)). Assuming that in a controlled environment, nitrous oxide and water vapor concentrations would remain constant, the output of the NDIR sensor was corrected for carbon dioxide concentration, which could change due to breathing in the lab, and used as the methane concentration (ppm). This methane value was compared against the TDLAS in the calibration. The INIR directly outputs methane concentrations in ppm.

### 2.6. Sensor Testing Method

One main concern with continuous monitoring sensors is the time taken for the sensors to respond to methane plumes. The time that the sensor is exposed to methane-enhanced air is a function of both the emission source and the characteristics of the wind. Some emissions last relatively short periods of time, e.g., blow-down events can occur over a single minute, while the direction of the wind field can vary in sub-minute time frames [6,35]. To investigate how the sensors in the WATCH_4_ERS units respond to high-frequency changes in emission, short-duration emissions will be run in a controlled environment and the lower-cost sensor response will be compared against the TDLAS sensor’s response.

Here, an experiment was conducted where 0.5% methane was released at 5 L per minute 0.5 m upwind of a WATCH_4_ERS unit (Supplementary Material Section S1 Figure S5). Airflow between the release point and the WATCH_4_ERS unit was controlled at 3.5 m s^−1^ using a fan blowing at a constant rate. Five experiments were conducted to investigate the response of the MOx, NDIR, INIR, and TDLAS sensors (Table 1). In each experiment, three gas plumes were released from a cylinder for a fixed duration with a controlled time between the plume releases. The aims of the experiment are to determine: 1. how many of the plumes could be seen in the instruments’ methane concentration data; 2. the typical time taken to respond to a downwind plume; and 3. the typical methane concentrations observed downwind.

## 3. Results

### 3.1. Sensor Calibration

**Figure 2:**
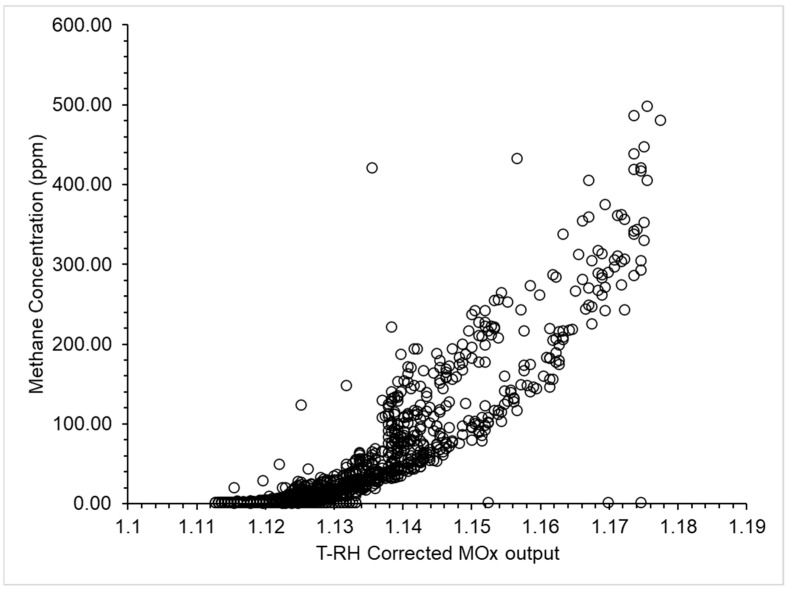
The metal oxide (MOx) temperature and humidity corrected output sensor output data plotted against the TDLAS methane concentration data (ppm). The MOx sensor voltage output, as measured by the potential divider circuit (Supplementary Material Section S1 Figures S1 and S2; with data capture described in Supplementary Material Section S3.1) was corrected for the effects of temperature and relative humidity and used to calculate the relative resistance of the metal oxide strip (*R_r_*, Supplementary Material Section S4 Equation (S1)). The R^2^ between the TDLAS and MOx data over the two hours of calibration was 0.77.

Following the calibration procedure described in Section 2.5, the sensor output data were plotted against the TDLAS methane concentration data to generate an understanding of the sensor’s response. The MOx data were converted from an output voltage and then corrected for the effects of temperature and relative humidity following published methods [19,20,27]. These corrected data show a logarithmic response to methane concentrations between background and 500 ppm with the sensor response saturating at concentrations greater than 300 ppm (Figure 2). The coefficient of determination of the regression (R^2^) between the TDLAS and MOx data over the two hours of calibration was 0.77. The scatter of points to the left of the logarithmic curve indicates a lag in the sensor’s response to changes in methane concentration.

**Figure 3:**
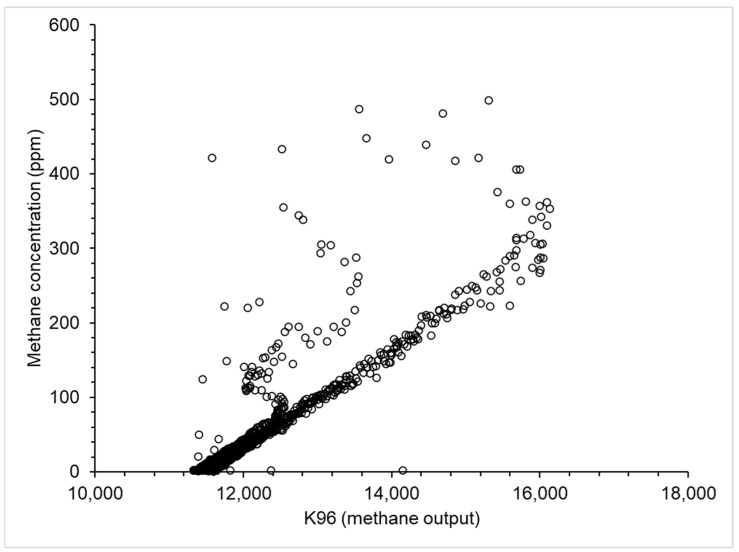
The non-dispersive infrared (NDIR) sensor output data plotted against the TDLAS methane concentration data (ppm). The output of the NDIR sensor was corrected for carbon dioxide concentration and used as a measure of the changing methane concentration. The R^2^ between the TDLAS and NDIR data over the two hours of calibration was 0.81.

The NDIR methane output data shows a linear response to changes in methane concentration between background and ~350 ppm. The scatter to the left of the regression line suggests a lag in NDIR response especially for the highest methane concentrations observed by the TDLAS. The coefficient of determination of the regression (R^2^) between the TDLAS and NDIR data over the two hours of calibration was 0.81 (Figure 3). The output of the INIR remained zero over the entire 2 h measurement period.

### 3.2. Sensor Testing

**Figure 4:**
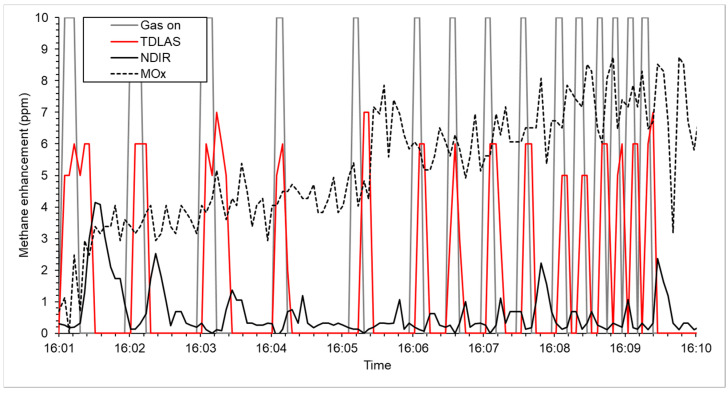
Sensor response (methane concentration, ppm) to methane released at 1 g CH_4_ h^−1^ released 0.5 m away and traveling at 3.5 m s^−1^. Sensors used were the metal oxide (MOx; black dashed line), Non-dispersion Infrared (NDIR; solid black line), and tunable diode laser absorption spectrometer (TDLAS, solid red line) sensors. The solid grey line, “Gas on”, shows when the gas was turned on (positive value) and tuned off (zero).

Following the calibration experiments, the sensors’ response was tested against short-duration plumes of methane. The TDLAS responded to the methane plumes within 5 s of the plumes being initiated 0.5 m away, note that the WATCH_4_ERS unit updates concentrations every 5 s (Figure 4). This suggests the TDLAS response is faster than the refresh rate of the WATCH_4_ERS unit. Reported methane concentrations were between 5 and 7 ppm for all experiments, which is in line with modeled values for a 1 g CH_4_ h^−1^ released 0.5 m away and traveling at 3.5 m s^−1^. The reported peaks also last the same amount of time as the emissions.

The NDIR sensor also responded to all methane peaks (Table 2). The response was between 12 and 18 s after the gas was released and methane peaks were lower than the TDLAS. The reported peaks from the NDIR lasted longer than the emission durations; however, the peak height at 1 g CH_4_ h^−1^ was sufficiently large to identify individual plumes. Individual plume peaks could not be distinguished in the MOx output even though the total methane observed over the experiment increased.

## 4. Discussion

The design, construction, and testing of the Wireless Autonomous Transportable Methane Emission Reporting System (WATCH_4_ERS), as described in this study, has resulted in the development of a sensing platform that can be used to measure methane across the scales of cost, precision, accuracy, and response rate. The WATCH_4_ERS uses four sensing technologies (MOx, NDIR, INIR, and TDLAS) to measure the methane concentrations in air at different upper/lower thresholds, with different levels of precision, and respond at different rates to changes in methane concentrations. Instrument response during typical calibration procedures and short-duration controlled release experiments presented here show the MOx, NDIR, and TDLAS sensors respond to changes in methane concentration and should be incorporated into future iteration of the WATCHERS unit. Initial findings from each sensor type’s response are presented below. The eventual goal of these units is to use multiple WATCH_4_ERS units at different distances from controlled methane release points in complex aerodynamic environments to 1. better understand the cost–benefit balance of methane sensors, and 2. identify how decreasing instrumentation costs could increase deployment coverage and therefore inform large-scale methane monitoring strategies.

Both calibration and response experiments indicate the INIR has little practical use for measuring methane concentrations less than 500 ppm. The INIR sensor was included as part of the WATCH_4_ERS instrumentation, as it has been reported that fast-responding sensors are able to distinguish high-concentration small-volume vortices within the larger plume [35] and it is currently unclear if sensors with higher detection limits, such as the SGX INIR, are fast enough to detect these. It is strongly suggested here that the INIR does not respond fast enough to be used as a downwind sensor.

The MOx sensor used here has been shown to have a logarithmic response to methane concentration change between background and 600 ppm. The previous literature suggests that response behavior is specific to individual sensors, with both linear and exponential responses reported [19,20,28]. Of note here is that the change in concentration during the calibration experiment was gradual (Figure 2) and the response of the MOx sensor passively sampling air, as presented here, can change over time fast enough to generate representative values for concentration measurement (Figure 3). The MOx response was found to be not fast enough when exposed to rapidly changing methane concentrations (Figure 4). It is strongly suggested here that a MOx mounted to passively sample air cannot respond fast enough to report emissions that either change in a sub-minute time frame or in variable winds. However, novel calibration methods [47], increased time averaging, or pumping air to the MOx sensors could overcome the issues and these sensors will continue to be tested as part of the WATCH_4_ERS unit.

The NDIR sensor reported linear change to methane concentration between background and 600 ppm, although there was a noticeable lag in reporting changing concentration, especially at higher values (>300 ppm). During the plume test, the NDIR responded to all plumes released and individual peaks could be observed throughout the experiment, even when the plumes were released 5 s apart. Our data suggest the NDIR takes between 12 and 18 s to respond to end plumes and the broad, lower peak reported indicates a slower overall response than the TDLAS sensor. The NDIR used here is also sensitive to other gases (N_2_O and CO_2_) and was tested in a controlled environment, so data output in a non-controlled environment may vary. However, given its costs, this passive instrument is a potentially useful sensor for long-term methane measurement.

The TDLAS unit has been shown to respond to changes instantly in methane concentration and is likely to be the most useful dataset generated by the WATCH_4_ERS unit for dispersion modeling. Despite this, the cost of the instrument makes it prohibitively expensive to deploy units at every facility emitting methane globally and an unrealistic option for widespread distribution. As such, the WATCH_4_ERS units will continue testing sensors to determine if operational modifications (e.g., pumps on the MOX sensors) can be installed or deployment locations optimized (e.g., INIR located nearer the sources) to better understand how lower-cost instrumentation can be used to detect, localize, and quantify methane emissions.

Future directions for this project include: 1. Incorporating sensors that use emerging technologies, e.g., those that use 2D materials, nanocomposites, or interband-cascade light-emitting technology [22,23,24,25,26]; 2. long-term stability tests; 3. real-world testing of emissions from all emissions sectors (energy, agricultural, waste, and natural); 4. investigating the potential impact of differing sensor update rates on the uncertainty and repeatability of sensing data, including data synchronization, processing, and storage; and 5. advanced calibration algorithms or sensor fusion techniques.

Additionally, we would like to investigate optimizing the rate at which air can be pumped onto the MOx strip to improve the sensor response time. Currently, we suspect that the delay in MOx response could either be a function of the resistance of the air movement to the metal oxide strip caused by the sensor structure, or a function of the time taken for the gas to be absorbed into the metal oxide strip and time taken for the resistance to change. Given the non-trivial nature of this task, we feel that this is a question that should be addressed following further measurements during a more detailed/focused study.

Gas selectivity is another interesting aspect of lower-cost sensor response, and will be explored during the real-world deployments when the WATCH_4_ERS units will be deployed downwind of sources from different emission sectors, i.e., agriculture or waste. The hypothesis is that sensors may only have utility for certain sources. For example, the MOx sensors are sensitive to other hydrocarbons and therefore of little use downwind of oil and gas production sites, while the NDIR is also sensitive to N_2_O which may impact the measurement of agricultural emissions.

## 5. Conclusions

This study presents a holistic description of sensor development, system incorporation, and programming code needed to integrate four methane sensing platforms (metal oxide sensors, a non-dispersive infrared sensor, an integrated infrared sensor, and a tunable diode laser absorption spectrometer) into a single measurement unit. Future work includes optimizing the WATCH_4_ERS for size so that they can be installed on mobile platforms and can be used to investigate spatial–temporal variability in methane concentrations.

## Figures and Tables

**Table 1:** Details of the five experiments conducted to investigate the response of the metal oxide (MOx), Non-dispersion Infrared (NDIR), integrated infrared (INIR), and tunable diode laser absorption spectrometer (TDLAS) sensors. Each experiment comprised three gas releases at a distance upwind of the sensors. “Distance” is the distance between the gas release point and the WATCH_4_ERS unit. “Emission rate” is the controlled emission rate of the gas (g CH_4_ h^−1^). The Duration is the length of gas releases. “Time between plumes” is the number of seconds between each gas release. As an example in experiment 5, gas was released at 1 g CH_4_ s^−1^ for 5 s, turned off for 5 s, on for 5 s, off for 5 s, on for 5 s, and then turned off.

Experiment #	Distance (m)	Emission Rate (g CH_4_ h^−1^)	Duration (s)	Time Between Plumes (s)
1	0.5	1	10	50
2	0.5	1	5	55
3	0.5	1	5	30
4	0.5	1	5	15
5	0.5	1	5	5

**Table 2:** The percentage of peaks observed (P), the average peak concentration (Av Peak), and the average time to detect the plume from initiation 0.5 m away.

	TDLAS			NDIR			MOx		
Expt	P	Av Peak (ppm)	Av Lag (s)	#	Av Peak (ppm)	Av Lag (s)	#	Av Peak (ppm)	Av Lag (s)
1	100	6.3	5	100	2.68	18	0	N/A	N/A
2	100	6.3	5	100	0.96	13	0	N/A	N/A
3	100	6.0	5	100	1.47	17	0	N/A	N/A
4	100	5.3	5	100	0.69	14	0	N/A	N/A
5	100	6.3	5	100	1.01	12	0	N/A	N/A

## Data Availability

Data can be accessed at the DRYAD research data repository (https://doi.org/10.5061/dryad.gf1vhhn0j accessed on 20 March 2025).

## Supplementary Materials

The following supporting information can be downloaded at: https://www.mdpi.com/article/10.3390/s25071954/s1. Section S1: Supporting Figures. Figure S1. Wiring for the Figaro TGS2600 and 2611 metal oxide sensors; Figure S2. Schematic of all USB connectors from the individual sensors attached to the USB hub and the hub attached to the laptop PC; Figure S3. Data capture from the ’46 Hawk tunable diode laser absorption spectrometer; Figure S4. Front view of Sensor box showing location of sensors; Figure S5. Downwind experiment side view; Section S2: Supporting Tables. Table S1. WATCH4ERS bill of materials; Table S2. Headers of the text files written as output by the “AMMMU_Python_Code.py” code; Section S3.1 TGS2600 and TGS2611—Data acquisition code; Section S3.1.1 Pseudo-code; Section S3.1.2 Arduino Code; Section S3.2 ’46 HAWK “Data sniffing” code; Section S3.2.1 Pseudo-code; Section S3.2.2 Arduino Code; Section S3.3 WATCH_4_ERS Python Code; Section S4 TGSXX Calibration.
