# Supplementary material for: Design, Build, and Initial Testing of a Portable Methane Measurement Platform
Source: Sensors (Basel). 2025 Mar 21;25(7):1954. doi: 10.3390/s25071954 (PMC11991200; doi:10.3390/s25071954)
Supplement: Supplementary file 1 [file sensors-25-01954-s001.zip › sensors-3481003-supplementary.pdf]

Design, build and initial tests of a portable methane measurement platform

Stuart N. Riddick[1,*], John C. Riddick[2], Elijah Kiplimo[1], Bryan Rainwater[1], Mercy Mbua[1], Fancy Cheptonui[1], Kate Laughery[1], Ezra Levin[1] and Daniel J. Zimmerle[1]

[1]Methane Emission Technology Evaluation Center (METEC), Energy Institute, Colorado State University, Fort Collins, CO 80524, USA

[2] Independent Researcher, Lockerbie, DG11 2BE, UK

***Correspondence:** stuart.riddick@colostate.edu

Supplementary Materials Section 1 – Supporting Figures

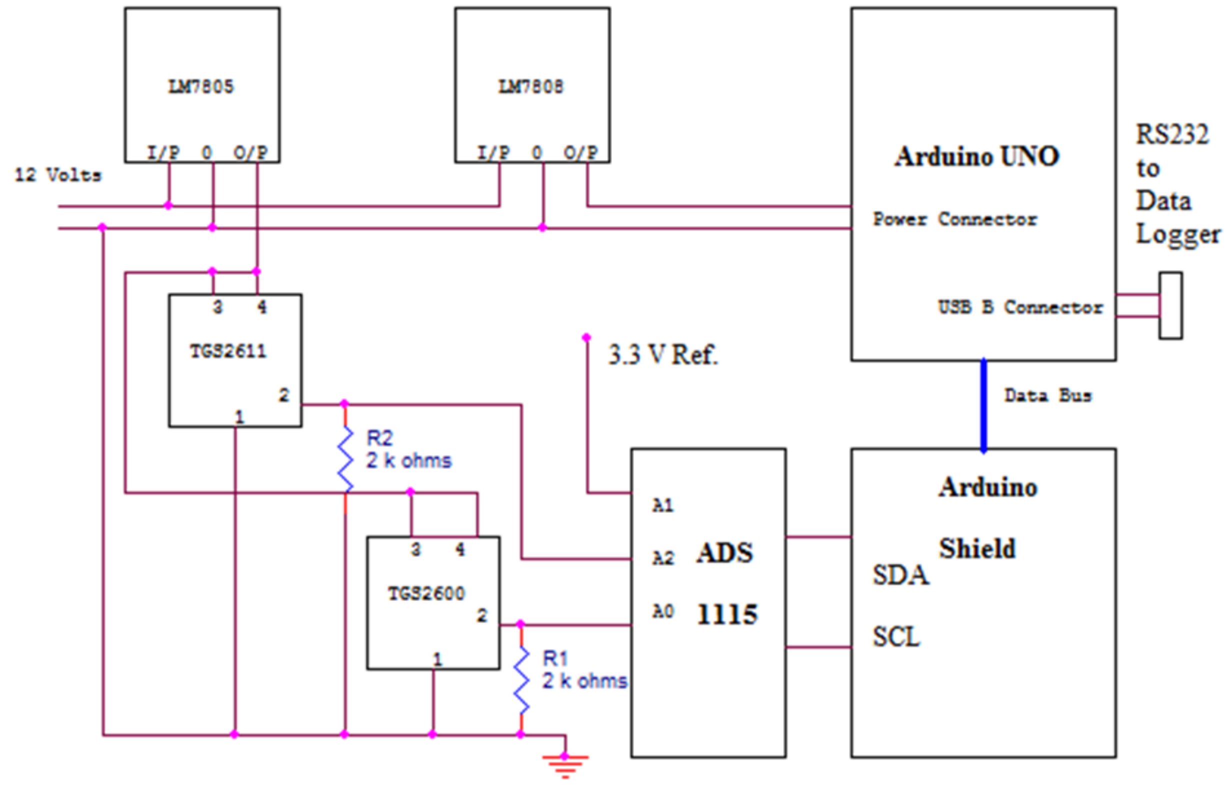

Figure S1. Wiring for the Figaro TGS2600 and 2611 metal oxide sensors.

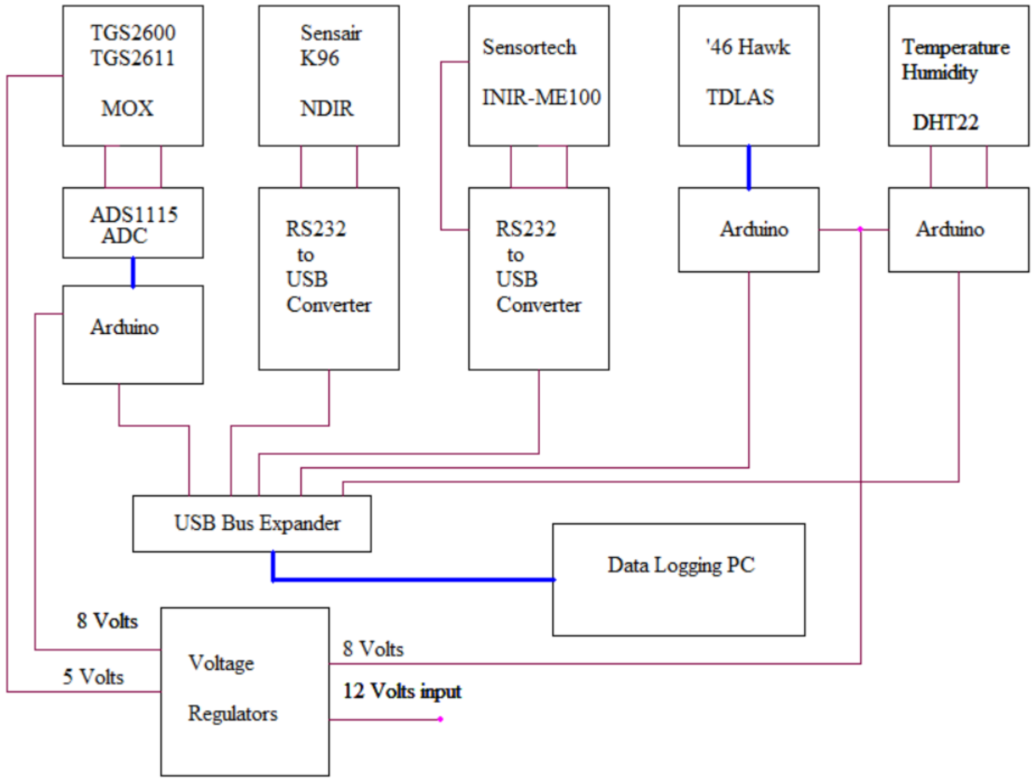

Figure S2. Schematic of all USB connectors from the individual sensors attached to the USB hub and the hub attached to the laptop PC.

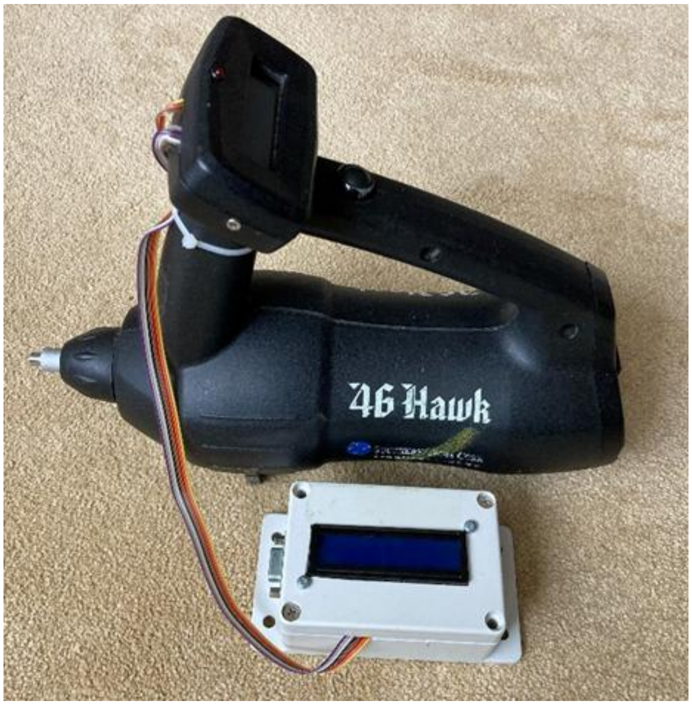

Figure S3. Data capture from the ’46 Hawk tunable diode laser absorption spectrometer.

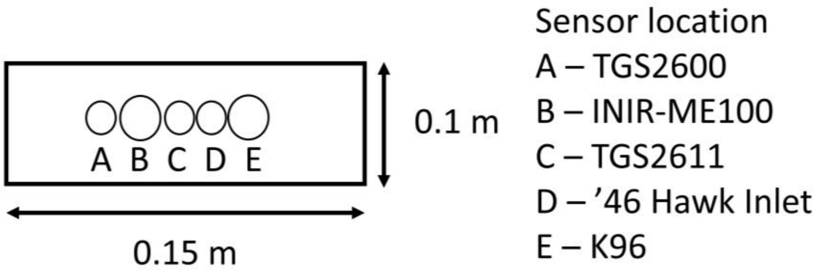

Figure S4. Front view of Sensor box showing location of sensors.

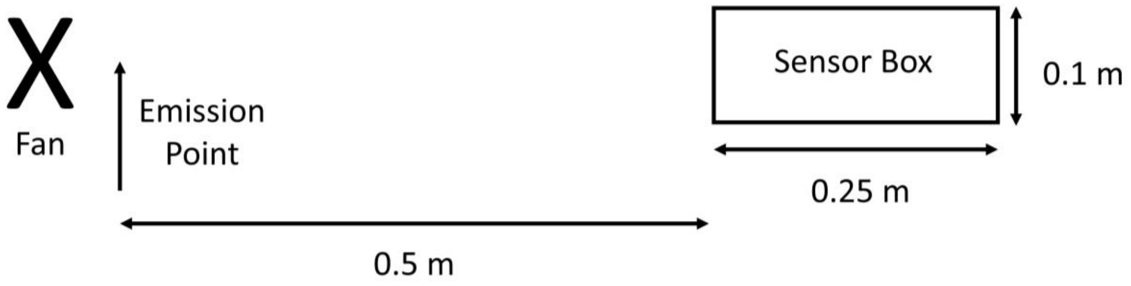

Figure S5. Downwind experiment side view.

Supplementary Materials Section 2 – Supporting Tables

Table S1. WATCH$_4$ERS bill of materials

Component	Number	Cost per unit (USD $)	Total cost (USD $)	Source of materials
Figaro TGS2600 sensor	1	20	20	www.figarosensor.com
Figaro TGS2611 sensor	1	20	20	www.figarosensor.com
SGX INIR-ME100 sensor	1	300	300	www.sgxsensortech.com
Senseair K96 NDIR sensor	1	500	500	www.senseair.com
Southern Cross Inc. ’46 Hawk	1	15,000	15,000	www. southerncrossinc.com
HP Elitebook 860 G10 Laptop PC	1	1000	1000	Digikey
DHT22 T-RH Sensor	1	10	10	Digikey
Arduino Uno REV 3	3	25	75	Digikey
ADS1115ADC Module	1	6	6	Digikey
DSD TECH USB to TTL Converter	2	20	40	Digikey
USB 2.0 to Serial Cable	2	10	20	Digikey
Powered USB hub	1	20	20	Digikey

Table S2. Headers of the text files written as output by the “AMMMU_Python_Code.py” code

Column	Header
1	HH:MM:SS
2	TGS2600 output (counts)
3	TGS2611 output (counts)
4	Reference output (counts)
5	K96 LPL (sum of CO_2, N_2O, and CH_4 concentrations in ppm)
6	K96 SPL (CO_2 concentration in ppm)
7	K96 MPL (H_2O concentration in ppm)
8	K96 Pressure from BME280 sensor (10 Pa)
9	K96 Temperature from NTC0 (0.01 °C)
10	K96 Temperature from NTC1 (0.01 °C)
11	K96 Temperature from ADuC MCU (0.01 °C)
12	K96 RH from BME280 sensor (0.01 %)
13	Hawk output (CH_4 concentration in ppm)
14	INIR output (CH_4 concentration in ppm)
15	INIR Temperature (°C)
16	DHT11 Relative Humidity (%)
17	DHT Temperature (°C)

Supplementary Materials Section 3 – Supporting Code

SM3.1 TGS2600 and TGS2611 – Data acquisition code

SM3.1.1 Pseudo-code

Line numbers at https://github.com/stuartnriddick/AMMMU.git/Riddick MOX Arduino Code.ino

```
Include necessary Arduino libraries (L9 to 17 &41)
Set specific variables (L18, 23, 39 &42)
Define variable types (L20 to 38)

Open Arduino setup (line 44)
        Begin serial, wire, Real Time Clock (RTC) & ADC (lines 46 to 48)
        Set Analogue Pin 1 as an input (line 50)
        Set pins 10 and 13 as output (L52 &53)
        Screen print ADC Range and change if required (L55 to 64)
        If RTC not working (L70)
                Screen print error message and turn on warning LED attached to pin 13 (L72 – 75)
        Reset Date and time using RTC (L76)
        If SC card writer not working (78)
                Screen print error message and turn on warning LED attached to pin 13 (L78 – 84)
        Delay 1s (L89)
        Reset date and time from RTC (L90)
End Arduino setup (L93)

Open program loop (L95)
        Clear data output string (L99)
        Reset date and time from RTC (L102)
        Generate a text data string using year month day hour minute second from RTC (L104 to 128)
        Define variables used to read ADC values (L137 &138)
        Read TGS2600 output from ADC pin 0 (L140)
        Read TGS2611 output from ADC pin 1 (L141)
        Convert values read by ADC to voltage (L142 &143)
        Add TGS voltages to text data string (L145 to 158)
        Run filename subroutine (L152)
                Generate a filename "LB"&YY&MM&DD&".txt" (L181 to 199)
        Open the file on the SD Card called ""LBYYMMDD.txt" (L153)
        If file is available
                Print text data string on screen(L157)
                Write text data string to file (L158)
                Close file (L159)
                Flash LED attached to pin 13(L162 &L172 to L179)
        Delay 2s (L169)
Loop to beginning of program loop (L170)
```

SM3.1.2 Arduino Code

```
// Program  Logger_B

// The data from the sensor is retrieved every
// 2 seconds and printed out.
// Time is read and SD card initialised
// Data are logged to SD file LByymmdd.txt
// Data logged are TGS and 3.3 volt reference 16 bit

#include <stdlib.h>
#include <SPI.h>
#include <SD.h>
#include <Wire.h>
#include "RTClib.h"
#include <Adafruit_ADS1X15.h>

Adafruit_ADS1115 ads;  /* Use this for the 16-bit version */
#include <SoftwareSerial.h>
const int chipSelect = 10;

char ID[2];
int a;
int b;
int c;
int d;
int Port;

int count_in;
int led = 13;
int Dummy;
int yy;
int Y20;
int mm;
int dd;
int h;
int m;
int s;
int i;
int old_sec;
int Flag =0;

RTC_DS1307 rtc;
char filename[] = "00000000.txt";

void setup() {
  Serial.begin(9600);
```

```
  Wire.begin();
 rtc.begin();
 ads.begin();

 pinMode(A1,INPUT);

 pinMode(led, OUTPUT);  // LED
 pinMode(10, OUTPUT);   // SD

  Serial.println("Getting single-ended readings from AIN0..3");
 Serial.println("ADC Range: +/- 6.144V (1 bit = 3mV/ADS1015, 0.1875mV/ADS1115)");

 // The ADC input range (or gain) can be changed via the following
 // functions, but be careful never to exceed VDD +0.3V max, or to
 // exceed the upper and lower limits if you adjust the input range!
 // Setting these values incorrectly may destroy your ADC!
 //

  ads.setGain(GAIN_ONE);        // 1x gain   +/- 4.096V  1 bit = 2mV      0.125mV

 delay(1000);
 delay(10);
 rtc.begin();
  if (! rtc.begin()) {
  //   Serial.println("Couldn't find RTC");
     digitalWrite(led, HIGH);   // LED ON indicating RTC problem
   while (1);
 }
  rtc.adjust(DateTime(F(__DATE__), F(__TIME__)));
// see if theSD card is present and can be initialized:
 if (!SD.begin(chipSelect)) {
  //  Serial.println("Card failed, or not present");

    digitalWrite(led, HIGH);   // LED ON indicating SD problem
   // don't do anything more:
   while (1);
 }
 // Serial.println("RTC Setup");
 // Serial.println("SD Setup");

 // Serial.println("Collecting Data");
 delay(1000);
 DateTime now =rtc.now();


}

void loop() {
```

```
 //  Data Sample Loop

 String dataString = "";  // Clear Output string

//  Read the time
DateTime now =rtc.now();

yy=now.year();

Y20 = yy-2000;
 dataString += yy;
 dataString += " ";
 mm=now.month();
 if (mm < 10) { dataString += '0';}
 dataString += mm;
 dataString += " ";
 dd = now.day();
 if (dd < 10) { dataString += '0';}
 dataString += dd;
 dataString += " ";
 h = now.hour();
 if (h < 10) { dataString += '0';}
 dataString += h;
 dataString += " ";
 m=now.minute();
 if (m < 10) { dataString += '0';}
 dataString += m;
 dataString += " ";
 s=now.second();
 if (s < 10) { dataString += '0';}
 dataString += s;
 dataString += " ";


 //dataString += " ";

 //  Read TGS  and store

  //Read data and store it to variables hum and temp

   int16_t adc0, adc1;
   float volts0, volts1;

adc0 = ads.readADC_SingleEnded(0);
adc1 = ads.readADC_SingleEnded(1);
volts0 = ads.computeVolts(adc0);
volts1 = ads.computeVolts(adc1);
```

```
    dataString += volts0;
    dataString += " ";
    dataString += volts1;
    dataString += " ";

// Write data to LByymmdd.txt

   getFilename(filename);
   File dataFile = SD.open(filename, FILE_WRITE);

  // if the file is available, write to it:
  if (dataFile) {
    Serial.println(dataString);
    dataFile.println(dataString);
    dataFile.close();
   // MQSerial.print(dataString);
   // MQSerial.print("\n\r");
     SMP_LED();  // flash sample LED
     SMP_LED();
    // print to the serial port and Bluetooth too:
    //   Serial.println(dataString);
    //    MONSerial.println(dataString);

  }
    delay(2000);
}

void SMP_LED()
{
 digitalWrite(led, HIGH);
 delay(50);
// Serial.println("Flashing LED");
 digitalWrite(led, LOW);
 delay(50);
}

void getFilename(char *filename) {
  int nyy;
   filename[0] = 'L';
   filename[1] = 'B';
   nyy = yy - 2000;
   filename[2] = (nyy)/10 + '0';
   filename[3] = nyy%10 + '0';
   filename[4] = mm/10 + '0';
   filename[5] = mm%10 + '0';
   filename[6] = dd/10 + '0';
   filename[7] = dd%10 + '0';
```

```
  filename[8] = '.';
  filename[9] = 'T';
  filename[10] = 'X';
  filename[11] = 'T';
//  Serial.println(filename);
   return;
  }
```

SM3.2 '46 HAWK "Data sniffing" code

SM3.2.1 Pseudo-code

Pseudocode for Sniffer_V7_9600_RS232 (Hawk - Arduino Interface)

Lines 9 -20 Set up Libraries and variables

Lines 21 – 33 ReadLcdBus1() Interrupt service routine called when LCD "E" goes low, data read from port D (8 bits) and Arduino digital input 8 are read and stored on fifo queue. Also increments errorCount id data errors detected.

Lines 34 – 117 Subroutine processQueue() called to reformat data stored on the queue, also checks if the LCD data is 4 bit or 8 bit data. In this version the data read from the LCD are always 4 bit data.

Lines 118 -129 Arduino Setup, interrupts initialised to be triggered on falling edge of LCD signal connected to Arduino digital input pin 2.

Line 130 – 185 Main data collection loop.

Lines 132 – 140 3 second loop to check if errorCount is zero, before starting to process the data.

Lines 141 – 162 Process any data on input queue checks the data are valid and stores data read from the LCD in the charBuffer (the output buffer).

Line 142 processQueue() called and returns the LCD data in the expandedBuf.

Lines 163 – 185 Checks if data are available in output buffer and formats them as HH Hawk data cr/lf before transmitting out to an Arduino serial port (Port A1).

SM3.2.2 Arduino Code

```
/*   Sniffer_V7_9600_RS232
  //   Data output as RS232 string 9600 baud on Port A
  //                                   |----- 4 bit ----|
  // Lcd              E   RS   RW    D7   D6   D5    D4
  // Arduino pin   2    3      4      5      6      7      8
  // Port            D2   D3   D4   D5   D6   D7   Pin 8
   */
#include "Fifo.h"
#include <SoftwareSerial.h>
SoftwareSerial DataSerial(A0,A1); // Rx A0, Tx A1
Fifo queue( 200 ) ;
volatile uint16_t errorCount = 0 ;  // indicates Queue overflows if the queue is not cleared fast enough in the loop()
char sBuff[80] ; // for sprintf
String datout;
// expandedBuf byte 0 is control information ( RS [bit1] and RW [bit0] from the LCD and an error flag [bit7]
// expandedBuf byte 1 This is LCD D7 to D0. In 4 bit mode, it is concatenated out of two 4bit nibbles.
uint16_t expandedBuf = 0 ;
void readLcdBus1() {
  // ports D and pin 8 are put on queue when LCD enable is falling.
  // called from external interrupt
  uint16_t buf = PIND ;
   buf <<= 8 ;
   int inB = digitalRead(8);
   uint8_t bufB = bufB + inB;
   buf  |= bufB;
```

```
  if ( ! queue.isfull() ) queue.push( buf ) ;
  else {
    errorCount ++ ; // queue overflow
  }
}
bool processQueue() {
  // takes an item off the queue, analyses it, reformats it and,
  // in the case of a 4 bit data stream, consolidates it
  // to form an 8bit packet. It returns true if the data item in
  // global expandedBuf is
  // complete (ie 8bits have been accumulated) and false otherwise.
  static bool mode8bit = true ;
  static bool highOrder4bit = true ;  // only valid in 4 bit mode
  bool expandedBufIsValid = false ;  // function return value
  uint16_t qItem = queue.pop_locked() ;
  // bulk read of ports
  uint8_t pinD = ( qItem >> 8 ) & 0xFF ;
  uint8_t pinB = ( qItem  ) & 0xFF ;
  // 8 low order bits of pinBuf are display pins D7 to D0
  uint16_t pinBuf = 0 ;
  bitWrite( pinBuf, 9  , bitRead( pinD, 3 ) ) ;   // RS
  bitWrite( pinBuf, 8  , bitRead( pinD, 4 ) ) ;   // RW
  bitWrite( pinBuf, 7  , bitRead( pinD, 5 ) ) ;   // D7
  bitWrite( pinBuf, 6  , bitRead( pinD, 6 ) ) ;   // D6
  bitWrite( pinBuf, 5  , bitRead( pinD, 7 ) ) ;   // D5
  bitWrite( pinBuf, 4  , bitRead( pinB, 0 ) ) ;   // D4
  bitWrite( pinBuf, 3  , bitRead( pinB, 1 ) ) ;   // D3
  bitWrite( pinBuf, 2  , bitRead( pinB, 2 ) ) ;   // D2
  bitWrite( pinBuf, 1  , bitRead( pinB, 3 ) ) ;   // D1
```

```
  bitWrite( pinBuf, 0  , bitRead( pinB, 4 ) ) ;   // D0

  bool functionSet8bit = false ;

  bool functionSet4bit = false ;

  // 4 bit function set recognised only in 8 bit mode

  functionSet8bit =  !( ( pinBuf & 0b0000001011110000 ) ^ 0b0000000000110000 ) ;  // RS = 0 and D7..D4
= 0011

  functionSet4bit =  !( ( pinBuf & 0b0000001011110000 ) ^ 0b0000000000100000 ) ;  // RS = 0 and D7..D4
= 0001

  // here we are interested in two possible situations

  // 1, We are in 8 bit mode and a Function Set 4 bit is encountered in the high order nibble or

  // 2. We are in 4 bit mode and a Function Set 8 bit is encountered in the high order nibble.

  // In the first case we change the mode to 4 bit and synchronise to the next nibble which will be a high
order nibble

  // and, according to the protocol, is another Function Set 4 bit command. The corresponding low order
nibble will be a screen

  // lines parameter and font size parameter.

  // In the second case we enter 8 bit mode then treat this as normal because nibble order
synchronisation is not relevant.

  if ( functionSet8bit && mode8bit ) {

    expandedBuf = pinBuf ;

    expandedBufIsValid = true ;

  }

  else if ( functionSet4bit && mode8bit ) {

    mode8bit = false ;

    highOrder4bit = true ;  // resync

    expandedBuf = pinBuf ;

    expandedBufIsValid = true ;

  }

  else if ( ! mode8bit ) {

    // 4 bit mode
```

```
    if (  highOrder4bit ) {
      // part 1 nibble
      if ( functionSet8bit ) {
        // transition to 8 bit
        mode8bit = true ;
        expandedBuf = pinBuf ;
        expandedBufIsValid = true ;
      }
      else {
        expandedBuf = pinBuf ;
        expandedBufIsValid = false ;
        highOrder4bit = false ;
      }
    }
    else {
      // part 2 nibble
      // join this with the first nibble
      expandedBuf = ( expandedBuf & 0b1111111111110000 ) | (( pinBuf & 0b0000000011110000 ) >> 4 )
;
      expandedBufIsValid = true ;
      highOrder4bit = true ;
    }
  }
  else if ( mode8bit ) {
    expandedBuf = pinBuf ;
    expandedBufIsValid = true ;
  }
  return expandedBufIsValid ;
}
```

```
void setup()
{
  Serial.begin(9600);
  Serial.println( "Starting Sniffer " ) ;
  DataSerial.begin(9600);
  pinMode(8,INPUT);
  pinMode(2, INPUT_PULLUP ) ;
  delay(2000);
  // LCD  "E" (arduino pin 2) is the Enable and we use the falling edge
  attachInterrupt( digitalPinToInterrupt( 2 ) , readLcdBus1 , FALLING ) ;
}

void loop()  // Data collection loop
{
  static uint32_t lastLoopAtMs = 0 ;
  if ( millis() - lastLoopAtMs > 3000 ) {
    // print out errors occasionally (if any )
    if ( errorCount != 0 ) {
      Serial.print( F("Q overflow error count= " )) ;
      Serial.println( errorCount ) ;
    }
    lastLoopAtMs = millis() ;
  }
  if ( ! queue.isempty_locked() ) {
    if ( processQueue() ) {
      // here if expandedBuf is ready to be read
      static char charBuffer[80 ] = {' '} ;      // for presenting a stream of characters obtained from the LCD
data bus.
```

```
    static uint8_t charBufferIndex = 0 ;

    bool isChar = ( ( 0b00000011 & ( expandedBuf >> 8 )) == 0b10 )  ; // RS == 1 and R/W = 0 (crude test
for a character)

    if ( isChar ) {

      char prCh ;

      uint8_t expBuf = expandedBuf & 0xFF  ;

      ( expBuf >= 0x20 && expBuf < 0x7F ) ? prCh = expBuf : prCh = '.' ;   // '.' => unrecognised

      snprintf(sBuff, 50, "control  data 0x%02X  0x%02X  \'%c\'", (expandedBuf >> 8) & 0xFF ,
expandedBuf & 0xFF , prCh  );

      if ( (uint16_t) (charBufferIndex + 1) < sizeof( charBuffer ) ) {

        charBuffer[ charBufferIndex++ ] =  prCh ;   // just the data part

        charBuffer[ charBufferIndex ] = 0 ;  // clean ahead

      }

    }

    else {

      snprintf(sBuff, sizeof( sBuff) - 1, "control  data 0x%02X  0x%02X ", (expandedBuf >> 8) & 0xFF ,
expandedBuf & 0xFF );

      if ( charBufferIndex > 0 ) {

        // dump charBuffer

        if(charBuffer[0] != 0x50){

          Serial.print("*");

          Serial.println( charBuffer ) ;

           String dataString = "";  // Clear Output string

        //  Load data

          dataString += 'H';

          dataString += 'H';

          dataString += ' ';

          dataString += charBuffer;

          dataString += '\n';

          dataString += '\r';
```

```
        DataSerial.println(dataString);
        }
        charBufferIndex = 0 ;
      }
    }
  }
 }
} // loop()
```

SM3.3 WATCH4ERS Python Code

```
      # Program Multi_4Chan_MQ4_K96_Hawk_INIR
      # Prog Version V3 Additional Temperature Arduino
      # Data are received automatically from INIR
      # Results are displayed on PC screen and written to File
      # Filename ID1,COMX,yrs,mths,dys.txt
      # V3 Lets the user to selectSensor ID
      # Data format ID1 Com port, Time INIR,Hawk
      # This version allows user to select individual Com Ports
      # Only one INIR device is written to and read from

from datetime import date
from datetime import datetime
import serial
import time
import sys
import signal
import binascii

def signal_handler(signal, frame):
      print("closing program")
      SerialPort.close()
      sys.exit(0)

SerialPort1 = serial.Serial("COM7",9600,stopbits=1,timeout=1.5) # MQ4
SerialPort5 = serial.Serial("COM3",9600,stopbits=1,timeout=5) # Temperature probe
SerialPort4 = serial.Serial("COM4",38400,stopbits=1,timeout=1.5) # INIR
time.sleep(4)

INIR_OutgoingData="[H]"  # Set INIR in Read/Demand Mode
SerialPort4.write(bytes(INIR_OutgoingData,'utf-8'))
time.sleep(1)

Flag = 0
while (1):
      try:
            SerialPort2 = serial.Serial("COM5",115200,stopbits=2,timeout=1.5) # K96
            OutgoingData= (0x68,0x04,0x00,0x00,0x00,0x08,0xf8,0xf5)  # Device Address 0x44

            #SerialPort1.write(bytes('S','utf-8'))
      except KeyboardInterrupt:
            print("Closing and exiting the program")
            #SerialPort1.close()
            sys.exit(0)
#  Read from MQ4 Sensor   *** Sort out Results
      SerialPort1.write(bytes('S','utf-8'))
```

```
        Flag = 0
        while(Flag == 0):
                InData = SerialPort1.readline().decode('utf-8').rstrip()
                Flag = 1
                #SerialPort1.close()

        x= InData.split()
        try:
                mq1 = (x[1])
                mq2 = (x[2])
                mq3 = (x[3])

        except:
                mq1 = -9999
                mq2 = -9999
                mq3 = -9999
        print('Got TGS')

#   Data collection  reads 20 bytes of K96 data
        SerialPort2.write(OutgoingData)
        Flag = 0
        line = SerialPort2.read(21)   # read 16
        a = (line[3]*256)+line[4]
        b = (line[5]*256)+line[6]
        c = (line[7]*256)+line[8]
        d = (line[9]*256)+line[10]
        e = (line[11]*256)+line[12]
        f = (line[13]*256)+line[14]
        g = (line[15]*256)+line[16]
        h = (line[17]*256)+line[18]
        SerialPort2.close()
        print('Got K96')

#  Get Hawk Data
        SerialPort3 = serial.Serial("COM6",9600,stopbits=1,timeout=5) # Hawk
        Indata = SerialPort3.readline()
        #print('Hawk ',Indata.decode('utf-8'))  # Test code
        In1 = Indata[2:10]
        #print(In1)    #Test code
        In2 = In1.lstrip()
        In3 = In2.rstrip()
        In4 = In3.decode('utf-8')

        try:
                Hawk = int(In4)
        except:
                Hawk = -9999
        SerialPort3.close()
```

```
        print('Got Hawk')

# Read Temperature probe
        #SerialPort5 = serial.Serial("COM19",9600,stopbits=1,timeout=5) # Temperature probe
        Indata = SerialPort5.readline().decode('utf-8')
        y = Indata.split()
        try:
            RH = (y[1])
            TTT = (y[2])
        except:
            RH = -9999
            TTT = -9999

        print('Got Temperature')

# Read INIR
        INIR_Cmd = "[Q]"
        SerialPort4.write(bytes(INIR_Cmd,'utf-8'))
        time.sleep(1)
        Flag = 0
        while(Flag == 0):

            IncomingData=SerialPort4.readline()   #  [
            if( IncomingData == b'\r0000005b\n'):  #THIS IS HEADER STRING
                Flag = 1
        In1=SerialPort4.readline() # Methane
        In2=SerialPort4.readline() # Error flag
        In3 = SerialPort4.readline()#Temperature
        In4=SerialPort4.readline() # Reference
        In5=SerialPort4.readline()
        In6=SerialPort4.readline()
        In7=SerialPort4.readline()

# Sort out input string to 3 individual samples
        if(In1):
            D2 = In1[1:9]
            T1 = In3[1:9]
            R1 = In4[1:9]
            #print(D2)   # Test code
            #print(T1)
# Convert data from Hex to dacimal

            try:

                D2I = int(D2,16)
                TT = (int(T1,16))/100
                Ref = int(R1,16)
            except:
```

```
            D2I = -9999
            TT = -9999
            Ref = -9999

    print('Got INIR',' ',D2I)
# Get PC time
    current_date = date.today()
    current_time = datetime.now()
    #print('Got time')   # Test code

# extracting the current year, month and day

    dys = current_date.day
    mths = current_date.month
    yrs = current_date.year
    yrs = yrs - 2000
    hh = current_time.hour
    mm = current_time.minute
    ss = current_time.second
#  Sorting out filenames and add 0 if < 10
    yrsa = str(yrs)
    mthsa = str(mths)
    if mths < 10:
        mthsa = '0'+ mthsa
    dysa = str(dys)
    if dys < 10:
        dysa = '0'+dysa

    fname1 = "MULTI_Sensor "+yrsa+mthsa+dysa+".txt"

    #print(fname1)   #  Test code

# Assign filename
    text_file1 = open(fname1,'a')

# Write results to file
    text_file1.write  (str(hh)+":"+str(mm)+':'+str(ss)+' '+str(mq1)+' '+str(mq2)+' '+str(mq3)+' '+str(a)+"
"+str(b)+" "+str(c)+" "+str(d)+" "+str(e)+" "+str(f)+" "+str(g)+" "+str(h)+" "+str(Hawk)+" "+str(D2I)+"
"+str(TT)+" "+str(RH)+" "+str(TTT)+ '\n')
    text_file1.close()
# Display results on PC
    print('Time ',str(hh),':',str(mm),':',str(ss),' ',str(mq1),' ',str(mq2),' ',str(mq3),' ',str(a),' ',str(b),' ',str(c),'
',str(d),' ',str(e),' ',str(f),' ',str(g),' ',str(h),' ',str(Hawk),' ',str(D2I),' ',str(TT),' ',str(RH),' ',str(TTT))

# Pause until next sample this value may require changing depending on output speed of INIR
    time.sleep(2)
```

Supplementary Materials Section 4 – TGSXX Calibration

Following the method of Eugster and Kling (2012) for the TGS2600, the relative resistance (R_r, Ω) was calculated from the sensor resistance (R_s, Ω), the sensor resistance in clean air (R_0, Ω), air temperature (T_a, °C), and the relative humidity (*rH*, %) (Equation S1; (Figaro, 2020; Riddick et al., 2020). Values for R_o taken as the minimum resistance since the power to sensor was last cycled (Riddick et al., 2020).

$$R_r = \left(\frac{R_s}{R_0}.(0.024 + 0.0072.rH + 0.0246.T_a)\right) \qquad \text{(Equation S1)}$$

For each sensor, R_r values were plotted against CH_4 mixing ratios measured by the TDLAS and a polynomial relationship generated to calculate the calibrated methane concentration to R_r. This polynomial relationship was then used to calculate a calibrated TGS26XX sensor methane mixing ratio. The regression coefficient (slope) and coefficient of determination (R^2) between the TDLAS and the TGS26XX sensor mixing ratios are presented as a measure of the TGS26XX sensor's ability to measure CH_4 mixing ratios.
